# Empowering mind-body wellness: effect of bundling seated exercises and psychoeducational rehabilitation using the teach-back approach on fatigue and coping of women postmastectomy

**DOI:** 10.1186/s12905-024-03242-5

**Published:** 2024-08-06

**Authors:** Zohour Ibrahim Rashwan, Samah Ramadan Shaheen, Ayah Shaban Abd-El Fattah Abd-El Rasoul, Neama Mohamed Fouad Kamel, Hamida Ahmed Mostafa Darweesh

**Affiliations:** 1 Pediatric Nursing, Faculty of Nursing, Alexandria University, Alexandria, Egypt; 2 Medical-Surgical Nursing, Faculty of Nursing, Alexandria University, Alexandria, Egypt; 3 Nursing Education, Faculty of Nursing, Alexandria University, Alexandria, Egypt; 4 Department of Nursing, College of Health and Sport Sciences, University of Bahrain, Zallaq, Kingdom of Bahrain; 5 Department of Nursing Sciences, College of Applied Medical Sciences, Prince Sattam bin Abdulaziz University, Wadi Addawasir, Riyadh, Kingdom of Saudi Arabia; 6 Community Nursing Department, College of Nursing, Imam Abdulrahman Bin Faisal University (IAU), Dammam, Kingdom of Saudi Arabia; 7 Nursing Department, Alriyada College for Health Science, Jeddah, Kingdom of Saudi Arabia

**Keywords:** Breast cancer, Exercise intervention, Psychoeducation, Teach-back; fatigue, Coping

## Abstract

**Background:**

Being diagnosed with Breast Cancer (BC) is a crisis that throws the patient’s life out of balance. Cancer-related fatigue is a debilitating sign experienced by women during and after BC treatment. Regular physical exercise may help mitigate patients’ fatigue, enhance coping abilities, improve their quality of life, and overall well-being. In parallel, psychological interventions are geared toward normalizing the lived painful experiences among oncology patients.

**Objective:**

to examine the effect of bundling seated exercises and psychoeducational rehabilitation using the teach-back approach on fatigue and coping of women postmastectomy.

**Methods:**

A quasi-experimental study was conducted in the Oncology Surgical Department and chemotherapy unit at the Alexandria Main University Hospital, Egypt. A total of 60 women were randomly allocated to either to the study or the control groups. Women in the study group practiced seated exercises and psychological rehabilitation interventions, including mindfulness breathing, problem-solving training, cognitive reframing technique, and thought stopping while the control group received the routine care.

**Results:**

The study revealed a significant decline in the fatigue mean scores among participants in the intervention group from 136.10 ± 27.76 to 98.43 ± 25.99 (*p* < 0.001). Similarly, there was a significant decrease in the patients’ mean scores of maladaptive coping, helplessness/ hopelessness (*p* = 0.014), and anxious preoccupation (*p* = 0.008). In contrast, there is a noticeable increment in the scores of adaptive coping, such as fighting spirit (*p* = 0.012), cognitive avoidance (*p* = 0.002), and fatalism (*p* = 0.009).

**Conclusion:**

Bundling seated exercises and psychological rehabilitation interventions using the teach-back approach have been proven to be simple and inexpensive non-pharmacological methods of reducing cancer-related fatigue and improving coping skills among women post-mastectomy.

**Trial registration number:**

NCT06360276, ClinicalTrails.gov, Retrospectively registered (April 8th, 2024), URL of trial registry record: https://clinicaltrials.gov/ct2/show/NCT06360276.

## Introduction

Breast cancer (BC) is a malignant condition characterized by the abnormal growth of the epithelial cells within the ducts or lobes of the breast tissue. It affects millions of women worldwide, comprising around one-third of cancers among women, and it is the second most common cause of female deaths [1]. In Egypt, there are over 22,000 newly diagnosed cases of BC among females annually, making it the most prevalent type of cancer, which represents approximately 33% of all cancer cases in women [2].

The management of BC depends on the pathological characteristics, receptor status, and cancer stage. The illness stage is influenced by multiple factors, including the tumor size, the number and location of affected lymph nodes, and the presence of metastasis [3]. Recently, there has been an evolution in BC management [4]. These advanced treatments include but are not limited to (a) modifications in surgical mastectomy procedures, (b) novel radiation therapy techniques, and (c) innovative systemic therapies for treating advanced BC and lowering the possibility of recurrence [5].

After mastectomy, patients may suffer from physical distress, deterioration of self-image, psychological anguish, sleep disturbances, and fatigue [6]. Fatigue is a universal symptom among patients with BC undergoing mastectomy. It is one of the devastating and poorly understood symptoms that may negatively affect the patients’ tolerance of the cancer treatment. Consequentially, it can be a significant reason for discontinuing or postponing their therapies [7].

The National Comprehensive Cancer Network (2020) defines cancer-related fatigue (CRF) as “a distressing, constant, individual sense of physical, emotional and/or cognitive tiredness or exhaustion related to cancer or cancer treatment that is not proportionate to recent activity and inhibits usual activity” [8]. The incidence of CRF among women with BC can vary significantly, with prevalence rates ranging from 25% to as high as 99%, depending on the treatment approach employed [9].

Unlike typical fatigue, CRF is more severe and less likely to be relieved by rest. It tends to worsen during the course of cancer management and may persist for up to five years after treatment completion. Fatigue, compared to all other side effects of cancer treatment, adversely impacts the general coping mechanisms and health-related quality of life among BC survivors [10]. Nurses play a vital role in managing CRF, where approximately 50% of patients with cancer prefer non-pharmacologic interventions [11].

Research studies demonstrated that physical exercises are practical non-pharmacologic interventions with substantial solid proof of therapeutic value in managing CRF [7, 10]. It is revealed that moderate-intensity exercise programs have a beneficial effect on physical fitness, behavioral and emotional parameters, and coping abilities among BC survivors [10]. These programs are a costless, secure, and efficient manner of offering long- and short-term psychological and physical benefits during BC treatment and recovery. It can help improve the strength of the arms, reduce shoulder joint stiffness, decrease neck and back pain, and thereby mitigate CRF. Accordingly, it enables the patients to resume activities of daily living and improves their overall health [7].

Being diagnosed with BC serves as a great source of stress. These patients may initially experience sudden collapses, become confused, and suffer from psychological problems, such as anxiety, depression, fear, and uncertainty about the future [12]. Therefore, BC survivors require assistance in adjusting to and satisfying their altered needs. Psychological rehabilitation, such as mindfulness breathing, thought-stopping, cognitive reframing, and problem-solving skills training have facilitated adaptation to BC. Such rehabilitation interventions are considered under the umbrella of cognitive and behavioral therapies to relieve patients’ emotional distress, foster optimistic appraisals, and build up their coping skills [13].

The teach-back strategy, also called the “show-me” technique or “closing the loop,” is one of the health educational strategies used to guarantee patient comprehension and promote patient-centered instructions. It entails asking the patients to repeat or re-explain health-related materials in their own words [14]. Patient education using the teach-back method has mutual responsibilities between educator and patient where the educator simplifies complex information, avoids medical jargon, and breaks down the teaching content into easily understood words. Then, the educator engages the patients by asking open-ended questions and attentively listening to their responses. Eventually, the educator identifies any misconceptions, knowledge gaps, or areas that require additional clarification in a nonjudgmental manner [15]. In the current study, the teach-back method offers a framework for instructing patients about seated exercise and psychological intervention. It empowers patients to actively participate in their care, follow treatment plans, and develop awareness about their health condition, available options, and self-care instructions [16].

Since, there are limited research studies exploring the effects of bundling physical and psychological interventions on clinical outcomes among women with BC, the interventions in this study are not only considered symptom management techniques but can also serve as rehabilitative tools for enabling women to recover from treatment adverse effects. By combining seated exercises with psychological rehabilitation interventions, the findings of this study have the potential to provide a holistic, supportive care approach for women with BC that addresses different aspects of their well-being.

## Aim

This study intended to examine the effect of bundling seated exercises and psychoeducational rehabilitation using the teach-back approach on fatigue and coping of women postmastectomy.

**Hypotheses for research**:

1. Women with BC who receive seated exercises and psychoeducational rehabilitation using the teach-back approach after mastectomy exhibit less fatigue than those who do not.
2. Women with BC who receive seated exercises and psychoeducational rehabilitation using the teach-back approach after mastectomy exhibit improved coping behaviors than those who do not.

## Methods

### Design and context of the study

This quasi-experimental, two groups, pre-posttest, longitudinal study was conducted at the Oncology Surgical Department and chemotherapy unit at the Alexandria Main University Hospital.

### Participants

A convenience sampling of sixty fully conscious adult women with BC underwent mastectomy. The sample size was calculated using the Epi info program used by power analysis according to the following parameters: population size 120, expected frequency = 50%, acceptable error 10%, confidence coefficient 95%, and minimum sample size of 53 patients.

**Fig. 1:**
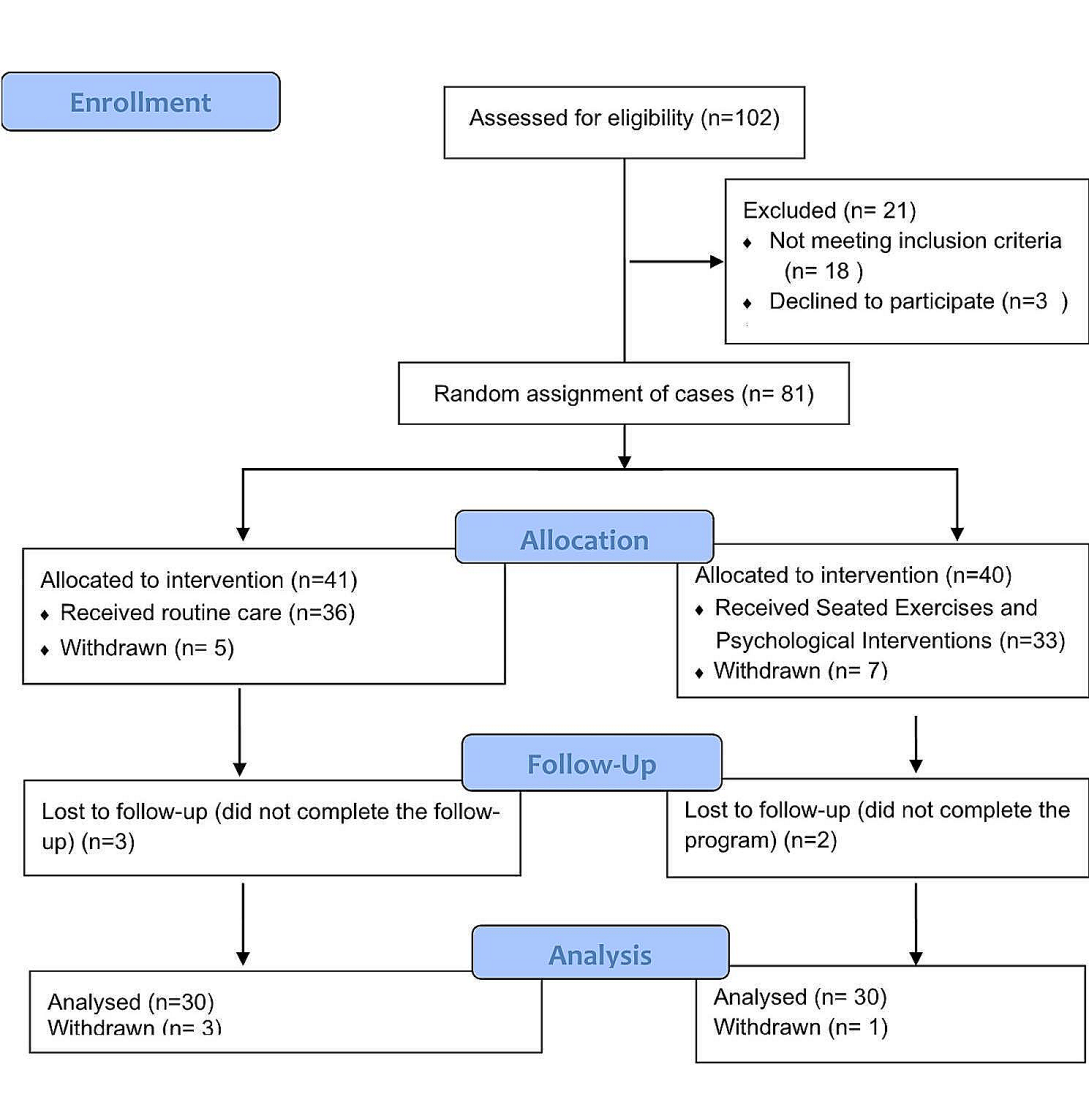
Flowchart of Patients’ Recruitment

During the study period (March 2021 to October 2021), 60 out of 102 patients with BC were eligible for the study. Participants were randomly divided into two equal parallel groups. (30 patients per group) utilizing a random number engine application, as illustrated in Fig. 1.

### Measurement tools

#### Piper fatigue scale (PFS)

The scale was initially developed by Piper et al. (1989) to measure the subjective dimension of fatigue [17]. It was revised by Piper B et al. (1998) and shortened from 42 to 22 items. The revised scale measures the four elements of subjective fatigue: behavioral/temporal (6 items), sensory (5 items), cognitive/mood (6 items), and affective/emotional meaning (5 items) [17]. Participants rated their level of fatigue on a scale ranging from 0 to 10. The total score ranges from 0 to 220; the higher the score, the higher the fatigue level (*r* = 0.98).

Questions regarding socio-demographic information of the patients (e.g., age, marital status, educational level, occupation, and income) as well as their clinical data (e.g., type of surgery, family history of cancer, past medical and surgical history) were attached to this tool.

#### Mini-mental adjustment to cancer scale (Mini-MAC)

It is a self-report survey created by Watson et al. (1994) and modified by Calderona et al. (2020) to gauge the cancer patients’ ways to cope [14, 18]. It consists of 29 items rated on a four-point Likert scale ranging from 1 “Does not apply at all to me” to 4 “Totally applies to me”). It includes five subscales under two main categories: maladaptive coping, including helplessness/hopelessness (8 items), and anxious preoccupation (8 items). However, adaptive coping includes fighting spirit (4 items), fatalism (5 items), and cognitive avoidance (4 items) [15]. According to Calderona et al. (2020, the examination of the critical elements indicates the presence of five variables, showing the construct’s validity, with good internal consistency in the subscales and Cronbach’s alpha values ranging from 0.78 to 0.93, and test-retest reliability, with r values ranging from 0.62 to 0.99) [14].

### Data collection procedure

**The data was collected through four phases as follows**:

**a. Assessment Phase**

Patients of both groups were initially assessed for their demographic characteristics, clinical data, fatigue, and coping behaviors.

**b. Preparation and Planning Phase**

As for the study group, the researchers prepared a health education unit about seated exercises and psychological interventions after thoroughly reviewing related literature [7, 16]. Educational objectives and content were also prepared. A booklet with real photos was designed as a tutorial aid. This booklet included breast anatomy, risk factors for BC, clinical manifestation, immediate postoperative and post-discharge care, seated exercises, and psychological rehabilitation. The educational materials were submitted to a panel of experts in the medical-surgical specialty (n-5) to assess the content validity. The overall agreement was 0.86 for relevancy and 0.91 for clarity.

#### Objective

This training aims to.

- Decrease patients’ cancer-related fatigue after mastectomy.
- Enhance patients’ abilities to control over their conditions.
- Empower the patient to adapt to the situation.

Teaching sessions for study group patients were planned according to their pre-scheduled time and place. The intervention was started at the preoperative period and continued to two months post-discharge/postoperative.

**c. implementation phase**

The established program was applied individually to each surgical patient and continued in the outpatient clinic and chemotherapy department.

The developed program about seated exercises and psychological interventions was conducted and applied individually to each surgical patient in the study group using the teach-back method.

The training was done by the researchers in 5 principles:

**Table:** 

Plan ahead	The researchers planned how they would request patients to teach back what they learned.
**Chunk and check**	The information/skill was broken down into smaller pieces for the patients, and they were asked to teach it back. This was repeated several times per session.
**Clarify and check again**.	In case of any misconceptions, the researchers clarified things and explained them in different ways, making sure the patients could appropriately articulate the topic on their own terms.
**Show-me**	The researchers asked the patient to re-demonstrate it in all practice sections.
**Use handouts**	The patients were given booklets and asked to highlight the most important instructions.

### Seated exercises

#### Session 1: Orientation lecture

The researchers first introduced themselves to the patients and explained the program’s general and specific objectives, timetable, and content. The session revolves around basic information about breast anatomy and the benefits of arm and shoulder exercises. Patients also received additional general instructions about arm and shoulder precautions, such as avoiding lifting heavy objects, receiving injections, blood draws, measuring blood pressure, and sleeping on the affected arm.

#### Session 2: Exercise demonstration and re-demonstration

In the sitting position, women were instructed to perform the following exercises. The researchers demonstrated each exercise to women and asked them to practice preoperatively to be ready for re-demonstrating them after surgery according to the timetable below;

**Table:** 

Type of exercise	Time of application
1. Active hand and elbow ROM exercise	1st day postoperative
2. Isometric hand and forearm exercise	2nd day postoperative
3. Active assistive and active flexion, abduction, internal and external rotation ROM exercise.	3rd day postoperative
4. Shoulder rolls, shoulder wings, arm circles, hands behind the neck, forward wall crawls, side wall crawls.	After drain removal

The patients were told to perform the exercise repeatedly until the researchers were sure that the patient had mastered the necessary skills.

### Psychological rehabilitation interventions

The participants received training about the implementation of four psychological interventions:

1. **Mindfulness breathing**: Patients are instructed to focus on breathing without allowing their minds to drift to the past or future. Patients were taught to sit comfortably and put one hand over the abdomen and the second on the chest. Then, take a deep breath, giving full attention to the passage of air from the nose to the lungs and to the oxygen in the air, feeding the body cells. Eventually, they are instructed to exhale from the mouth, as if expelling worries and sorrows in that exhalation to get rid of worries and sorrows by choosing a calming focus on the manner of their breathing only. Patients were instructed to repeat this twice a day and make it a lifestyle, and they will feel the difference.
2. **Problem-solving training** (PST) helps individuals gain a range of skills such as information seeking, planning, taking direct action, seeking social support, evaluating the pros and cons, and learning new skills that help them cope more effectively with life-threatening conditions like cancer. It also trains them to deal with the cause of their problems and to change or eliminate the source of their stress. The problem-solving process involves seven steps: identifying the exact issue, establishing an objective, determining the root cause of the problem, creating an action plan, carrying out the action plan, assessing the results, and constantly improving.
3. **Cognitive reframing technique**, in which patients are told that “how one thinks can affect how one feels.” For instance, patients were taught to use the “ABC Thinking Model” (where “A” = the activating or triggering event, “B” = a given belief, attitude, or point of view, and “C” = the emotional effect based on that belief relative to “reality”) to decide if those negative beliefs need to be modified.
4. **Thought Stopping**: Stopping the thinking process means first noticing a negative, nervous, or unhelpful thought. Whenever a client realizes they are thinking about the target, they say “stop” or interfere somehow.

Eventually, the researchers distributed the booklet that included all the instructions mentioned (seated exercises and psychological rehabilitation) with a detailed explanation of each activity and illustration pictures to serve as a manual guide at home.

### Follow-up

Women were instructed to practice each exercise ten times and repeat the whole activity five times per day as guided by the booklet. Women were followed up for two months by conducting daily communication through social networking applications and telephone calls to ensure their commitment to the program and provide the needed support. The researchers also met the participants at outpatient clinics and the chemotherapy department, emphasizing the daily practice of the interventions.

**The control groups** received routine health education in the unit, where nurses provided general instructions about the postoperative activities for women with BC.

d. evaluation phase

Patients of the two groups were re-evaluated after two months for their fatigue and coping using tools I and II.

### Ethical considerations

Ethical approval was obtained from the Ethical Research Committee at the Faculty of Nursing, Alexandria University, to conduct the study (IRB: 14/1/2021), and the study was retrospectively registered on clinicalTrails.gov (NCT06360276). Additional permission was obtained from the responsible authorities for the study setting after explaining the study’s aim. Before applying the interventions, the eligible patients received a detailed explanation of the aim, benefits, and any possible risks of the study. The researchers emphasized voluntary participation and the right to withdraw from the study at any time. Participants’ privacy and anonymity, as well as confidentiality, were ascertained. Accordingly, written informed consent was obtained.

### Statistical analysis

Statistical Package for Social Sciences (SPSS) version 23.0 was used. Descriptive statistics were used to describe the socio-demographic characteristics of women, their past medical and surgical history and lifestyle, mean fatigue scores, and adjustment to cancer. After testing normality, the Mann-Whitney Test was used to compare the total mean scores of women’s fatigue and adjustment to cancer. In contrast, Chi-square or Fisher’s Exact tests were utilized to compare the socio-demographic characteristics of women, their past medical and surgical histories, and lifestyles. At *P* *≤* 0.05, all statistical analyses were deemed significant.

## Results

Table 1 shows that more than half (56.7% and 60.0%, respectively) of patients in the study and control groups aged 40 to 60 years old have primary education (50.0% and 40.0%, respectively). The majority (70.0% and 83.3%, respectively) of both groups were married. Furthermore, more than half (56.7%, 53.3%, respectively) were non-employed (63.3%, 66.7%, respectively) urban residents.

**Table 1:** Women Socio-demographic characteristics

	Socio-demographic data	Study Group (*n* = 30)	Control Group (*n* = 30)		Test of Sig.	*p*
		No. (%)		No. (%)		
Age	20-<40	13(43.3)		12(40.0)	**χ**^**2**^ **=** 7.065	0.072
	40–60	17(56.7)		18(60.0)		
Education	Illiterate	6(20.0)		10(33.3)	F^ET=^ 8.103	0.107
	Basic Education	15(50.0)		12(40.0)		
	High School	7(23.3)		8(26.6)		
	Bachelor	2(6.7)		0(0.0)		
Marital Status	Single	3(10.0)		4(13.3)	F^ET=^ 3.954	0.183
	Married	21(70.0)		25(83.3)		
	Divorced	6(20.0)		1 (3.3)		
Occupation	Employed	13(43.3)		14(46.7)	χ2 = 4.51	0.176
	Non employed	17(56.7)		16(53.3)		
Residence area	Rural	11(36.7)		10(33.3)	**χ**^**2**^ **=** 1.073	0.787
	Urban	19(63.3)		20(66.7)		

**F**^**ET**^**Fisher Exact Test** χ^2^: **Chi square test** *: Statistically significant at *p* ≤ 0.05

Table 2 showed that nearly one-third (30.0%, 36.7%, 33.3%, 33.3%, 36.7%, and 26.7%, respectively) of both groups underwent a radical or modified radical mastectomy. Most of both groups have had previous hospitalizations, and hypertension was the main reason for admission. Most of the study groups have no history of previous surgery, while the majority of the control groups have. Furthermore, most have a family history of BC related to their mothers, with no previous exposure. The table also displayed no significant differences between groups regarding their past medical and surgical histories and lifestyles.

**Table 2:** Comparison between the two studied groups according to past medical and surgical history and lifestyle

Past Medical and surgical history and lifestyle	Study Group (*n* = 30)		Control Group (*n* = 30)		Test of Sig.	*p*
	No.	%	No.	%		
**Current Surgery**					**F**^**ET=**^ 1.264	0.895
Lumpectomy	0	0.0	1	3.3		
Mastectomy	9	30.0	10	33.3		
Radical mastectomy	11	36.7	11	36.7		
Modified radical mastectomy	10	33.3	8	26.7		
**Previous Hospitalization**	12	40.0	21	70.0		
**#Reason for admission**	**(** ***n*** ** = 12)**		**(** ***n*** ** = 21)**			
Hypertension	9	75.0	13	61.9	^FE^*p*=0.703	
Diabetes Mellitus	4	33.3	11	52.4		0.290
Anemia	1	8.3	0	0.0		
**Previous Surgery**	12	40.0	20	66.7		
**History of Cancer**						
No	26	86.7	25	83.3	**F**^**ET**^ =3.288	0.238
Breast cancer	4	13.3	2	6.7		
Ovarian cancer	0	0.0	3	10.0		
**Family History of BC #**						
Mother	13	72.2	12	60.0	**F**^**ET**^ =3.811	0.171
Sister	5	27.8	4	20.0		
Grandmother	0	0.0	4	20.0		

χ^2^: **Chi-square test F**^**ET**^: **Fisher Exact Test**

#: Categories are not mutually exclusive

Table 3 showed a highly significant difference between all fatigue dimensions among patients with mastectomy at pre and post-test, whereas *p* < 0.001.

**Table 3:** Comparison between fatigue dimensions among patients with mastectomy at pre and posttest

Fatigue Dimensions	Pretest		Sig.	Posttest		Sig.
	Intervention Group	Control Group		Intervention Group	Control Group	
	Mean ± SD	Mean ± SD		Mean ± SD	Mean ± SD	
Behavioral Severity	6.94 ± 1.61	7.11 ± 1.68	Z^MW^= -0.82 *p* = 0.41	4.76 ± 1.23	6.85 ± 1.10	Z^MW^= -5.04 *p <* 0.001
Affective / meaning	6.67 ± 1.68	6.91 ± 1.79	Z^MW^= -0.73 *p* = 0.47	5.11 ± 1.65	5.68 ± 1.17	Z^MW^= -2.39 *p* = 0.02
Sensory	6.55 ± 1.62	6.20 ± 1.56	Z^MW^= -1.17 *p* = 0.24	3.31 ± 1.63	6.43 ± 1.84	Z^MW^= -4.77 *p <* 0.001
Cognitive	6.68 ± 1.56	5.52 ± 1.41	Z^MW^= -3.06 *p* = 0.12	4.63 ± 1.14	5.74 ± 1.61	Z^MW^= -3. *p <* 0.001

Z^MW^: **Mann Whitney Test Significant at**^**b**^***p*<0.01**, ^**C**^***P*<0.001**

**Fig. 2:**
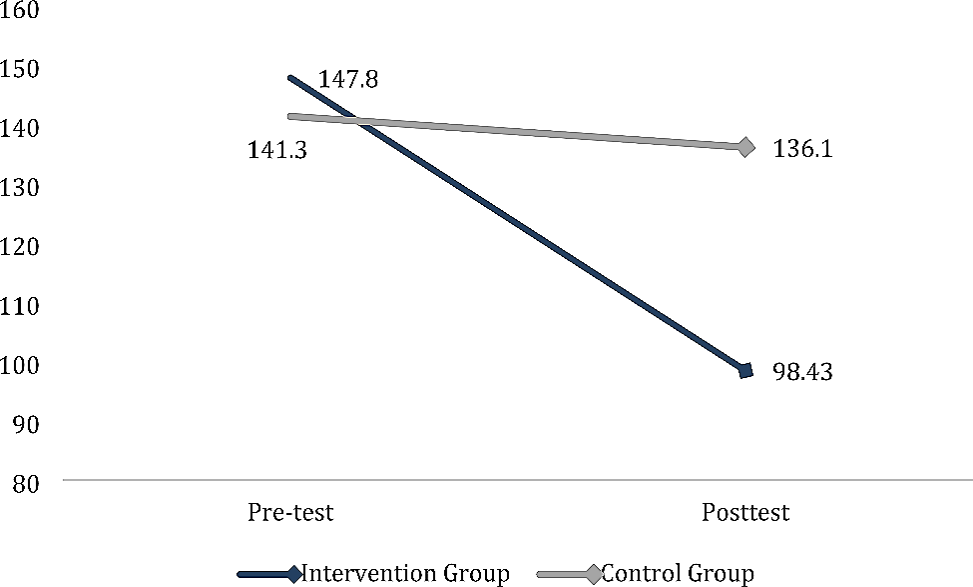
Comparison between Total Scores of Fatigue among patients in the intervention and control groups at pre and posttest

Figure 2 revealed that women in both groups had high levels of fatigue at the pretest as the total mean scores were 147.80 ± 34.75 for the intervention group and 141.30 ± 34.07for the control group with no significant statical differences between the two groups (*p* = 0.183). There was also a significant reduction in the total fatigue score after the implementation of seated exercises using the teach-back method, where the mean scores reduced from 136.10 ± 27.76 to 98.43 ± 25.99 ( *p* < 0.001).

In relation to maladaptive coping behaviors, Table 4 showed that there was no statistically significant difference between the intervention and control groups in the pretest (*p* = 0.87) in relation to the feeling of helplessness or hopelessness dimension, while a statistically significant difference was found between the two groups in the post-test. As for the anxious preoccupation dimension, a statistically significant difference was found between the intervention and control groups in the pretest and post-test, respectively (*p* < 0.001, *p* = 0.008). Regarding the adaptive coping behaviors, the table showed that there was no statistically significant difference between the intervention and control groups in the pretest in relation to fighting spirit, cognitive avoidance, and fatalism.

**Table 4:** Comparison between Adjustment to Cancer Scale among patients with mastectomy at pre and posttest

	Pretest		Sig.	Posttest		Sig.
	Intervention Group	Control Group		Intervention Group	Control Group	
	Mean ± SD	Mean ± SD		Mean ± SD	Mean ± SD	
1. **Maladaptive Coping**						
Helplessness/ Hopelessness	3.36 ± 0.60	3.55 ± 0.25	Z^MW^= -0.17 *p* = 0.87	2.83 ± 0.51	2.52 ± 0.49	Z^MW^= -2.46 ***p*** **= 0.014**^**b**^
Anxious Preoccupation	3.24 ± 0.37	3.22 ± 0.33	Z^MW^=-3.59 *p* = 0.00	2.59 ± 0.50	2.94 ± 0.49	Z^MW^=-1.74 ***p*** **= 0.008**^**b**^
**2.** **Adaptive Coping**						
Fighting Spirit	2.52 ± 0.59	2.49 ± 0.52	Z^MW^=-1.64 *p* = 0.101	3.08 ± 0.38	2.66 ± 0.59	Z^MW^=-2.53 ***p*** **= 0.012**^**a**^
Cognitive Avoidance	2.53 ± 0.48	2.68 ± 0.60	Z^MW^=-2.68 *p* = 0.071	3.19 ± 0.61	2.70 ± 0.25	Z^MW^=-3.16 ***p*** **= 0.002**^**b**^
Fatalism	2.19 ± 0.28	2.38 ± 0.68	Z^MW^=-0.93 *p* = 0.352	3.07 ± 0.46	2.72 ± 0.51	Z^MW^=-2.61 ***p*** **= 0.009**^**b**^

## Discussion

Being diagnosed with BC is a crisis that throws the patient’s life out of balance. Cancer is one of the particularly stressful life conditions. As the disease develops, patients face several psychological symptoms, such as low self-esteem, disturbed body image, and dying anxiety [19]. Fatigue is a typical debilitating sign experienced by women with BC during and after therapeutic treatment. Exercise and psychological interventions are known as excellent non-pharmacological interventions that increase the patients’ coping abilities and overall psychological well-being [8]. So, designing an empowering self-management program using a teach-back approach enables patients to cope with their new situation [13, 20, 21].

The present study findings showed a significant reduction in CRF after the implementation of seated exercises before and after the intervention. The physiological mechanisms by which exercise helps mitigate fatigue are not fully understood, but it is thought to involve improvements in cardiovascular fitness, muscle strength, and energy metabolism. Those changes are likely to be similar to those that boost mood, such as releasing hormones, neurotransmitters, and other substances during and after exercise [22]. Exercise also impacts sleep patterns, which has a knock-on effect on exhaustion and energy levels. When patients exercise, they sleep better and feel more energized [23, 24]. Another probable cause of reducing CRF is enhanced physical performance, which may raise patients’ independence and sense of control and foster self-confidence. Social interaction and engagement in physical activity might improve one’s mood, self-esteem, and coping skills [25].

Several studies supported these findings [10, 26, 27]. These studies asserted that exercise is safe and helpful in improving CRF and overall quality of life among patients with BC. Besides, Song et al. (2021) concluded that supervised exercise reduces CRF and should be employed in BC rehabilitation settings [28]. Furthermore, the current study’s findings are consistent with Barahoui et al. (2021) and Shahandashti et al. (2021), who concluded that teach-back-based training could reduce fatigue, pain intensity, improve sleeping patterns, and increase self-efficacy in patients with BC [21]. Thus, the use of teach-back-based training in the care of these patients is recommended. Furthermore, Özkan et al. (2022) reported that supervised combined exercise training during adjuvant radiation lowered anxiety and weariness whilst it invigorated energy, amended general health perceptions, and prevented a decline in effective coping methods [25].

The results of the present study showed a significant reduction in all maladaptive coping behavior level dimensions, namely, helpless/hopeless and anxious preoccupation among patients in the study group compared. Furthermore, there was a noticeable improvement in the patients’ adaptive coping behavior, including fighting spirit, cognitive avoidance, and fatalism in patients in the intervention group. The results of the present study could be justified in light of the fact that psychological rehabilitation included a wide variety of techniques to enhance the patient’s coping mechanisms. For instance, mindfulness breathing enabled patients with BC to expel their worries and sorrows and choose a calming focus [22]. The program also focused on the patient’s cognitive abilities as the researchers trained the participants to perform cognitive reframing techniques, problem-solving, and thought-stopping [29]. Taken together, these would equip the patients with a range of skills that empower them to cope effectively with such emergent life-threatening conditions and discontinue negative, unhelpful thoughts [12, 13]. The current study’s findings are consistent with previous studies, which highlighted that the intervention group’s mean adaptive coping score was significantly higher than the control group’s. These studies also discovered that the psychological educational intervention program increased the research group’s adaptive coping abilities while decreasing maladaptive coping practices [22].

A similar study conducted by Younis et al. (2020) also emphasized the benefits of psycho-educational intervention programs in increasing patients’ awareness of their own emotions, which influence their coping process [30]. Furthermore, the findings of Cipolletta et al. (2019) suggest that psycho-educational support interventions for oncology patients may be helpful in comparing various strategies in mitigating the lived stressful experiences [29]. They appear to improve coping mechanisms, self-efficacy, social functioning, perceptions of well-being, and quality of life while minimizing their distress and anxiety [29]. Furthermore, Gong et al. (2022) revealed that structured psychological care and teach-back health education could dramatically reduce sorrow and fear in the intervention group while improving the use of coping mechanisms [31]. The teach-back technique allows patients to communicate their understanding of the teaching material in their own words, making it more effective than traditional methods of instruction. When patients understand what is going on with their bodies and the mechanics of a procedure, they feel more at peace and are less inclined to worry [32].

### Strengths and limitations

Even though the current study showed a favorable impact of the intervention bundle, the small size of the sample used in the research may affect the generalizability. So, further studies with larger sample size are required to confirm the findings. Using convenient sampling was another limitation that was overcome by random assignment of the cases to the study and control groups. Moreover, a low educational level hindered the patients’ compliance with the given instructions. The- researcher also struggled with the patients’ dropouts of completing the program’s sessions. Therefore, the researchers faced the challenge of simplifying and tailoring the teaching content according to the participants’ different learning styles to boost their understanding. The use of the teach-back strategy is one of the strengths that simplified the given instructions and helped the patients practice the complex skills step-by-step. Moreover, the study’s interventions were directed to enhance multiple aspects of the patient’s health condition, including physical, psychological, and cognitive.

## Conclusion

Bundling seated exercises and psychological rehabilitation interventions using the teach-back method have been proven to be a simple and inexpensive non-pharmacological method of reducing fatigue and improving coping among women with BC.

## Data Availability

The datasets used and/or analyzed during the current study are available from the corresponding author on reasonable request.
